# A Qualitative Study of the Values, Needs, and Preferences of Patients Regarding Stroke Care: The ValueCare Study

**DOI:** 10.5334/ijic.6997

**Published:** 2023-07-17

**Authors:** Esmée L. S. Bally, Demi Cheng, Amy van Grieken, Dianne H. K. van Dam-Nolen, Stefania Macchione, Mireia Ferri Sanz, Áine Carroll, Bob Roozenbeek, Diederik W. J. Dippel, Hein Raat

**Affiliations:** 1 Department of Public Health, Erasmus MC University Medical Center, Rotterdam, The Netherlands; 2 Department of Neurology, Erasmus MC University Medical Center, Rotterdam, The Netherlands; 3 European Project Office Department, Istituto per Servizi di Ricovero e Assistenza agli Anziani (Institute for Hospitalization and Care for the Elderly), Treviso, Italy; 4 R&D+i Consultancy, Kveloce I+D+i (Senior Europa SL), Valencia, Spain; 5 School of Medicine, University College Dublin, Dublin, Ireland; 6 Academic Department, National Rehabilitation University Hospital, Dublin, Ireland

**Keywords:** stroke patients, values, needs, experiences, value-based health care, integrated care

## Abstract

**Introduction:**

An in-depth understanding of patient perspectives contributes to high-quality, value-based health care. The aim of this study was to explore the values, needs, and preferences of stroke patients across the continuum of care.

**Methods:**

We performed a qualitative study, as part of the larger ValueCare study, involving 36 patients who have had ischemic stroke within the past 18 months at the time of recruitment. Data were collected between December 2020 and April 2021 via one-to-one telephone interviews. All interviews were audio-taped and transcribed verbatim. The interview data were analysed using a thematic approach.

**Results:**

The analysis resulted in five themes: (1) patients’ values about health care, (2) information and education, (3) psychological support, (4) follow-up care, and (5) continuity and coordination of care. Patients valued a compassionate professional who is responsive to their needs. Furthermore, patients indicated a need for tailored health information, psychosocial services, pro-active follow-up care and improved coordination of care.

**Discussion and conclusion:**

Stroke patients emphasised the need for tailored information, psychological support, pro-active follow-up, and improved coordination of care. It is advocated for professionals to use a value-based care approach in order to satisfy the individual needs of patients with regard to information, communication, and follow-up care.

## 1. Introduction

Each year approximately 1.12 million European citizens have a stroke [1], of which 85% are ischemic strokes [2]. Due to population ageing and improved survival rates, the number of people living with stroke is projected to rise from 9.53 million in 2017 to 12.11 million in 2047 – an increase of 27% [2,3]. Despite advancement in medical care, stroke remains a leading cause of disability [4]. Patients can experience long-term difficulties in terms of physical impairments, social reintegration and emotional functioning [5,6,7]. Post-acute stroke care aims to address these social and functional determinants of recovery including access to ongoing diagnostics, therapy, rehabilitation, psychological support, and self-management strategies [8].

Stroke care consists of three phases: (i) acute care, (ii) rehabilitation, and a (iii) chronic phase of long-term support [9]. Usually, a patient journey begins with the timely recognition of and response to symptoms, followed by rapid diagnosis and reperfusion (if appropriate), and measures to prevent complications [10]. In most high-income countries, care at a stroke unit is the central feature of modern stroke service [11]. This includes early rehabilitation activities to advance discharge home from the hospital. Early supported discharge teams have the potential to link hospital care and community-based care to support ongoing rehabilitation [12]. The Action Plan for Stroke in Europe recommends providing a documented plan for community rehabilitation and self-management support for all stroke patients [8].

The involvement of various disciplines, institutions and organisations in stroke care, such as hospitals, rehabilitation centres, nursing homes, general practices, and home care providers, could complicate the care process of this patient group. Stroke care providers in the Netherlands are reimbursed using a fee-for-service system; each care provider receives separate reimbursement [13]. Fee-for-service systems lack incentives to improve patient transitions and coordination of care. Stroke services are, therefore, best positioned in regional partnerships that encourage integrated care among all care providers [14]. This includes processes of linking and coordinating services to overcome fragmentation. Value-based health care can support this process by reorganising care delivery around a patient’s medical condition. ‘Value’ in value-based health care can be defined as health outcomes achieved relative to the costs of care [15]. By bundling the costs of all services delivered to a patient across the continuum of care, bundled payments create a financial incentive for providers to quality and efficiency of care [16].

**Figure 1:**
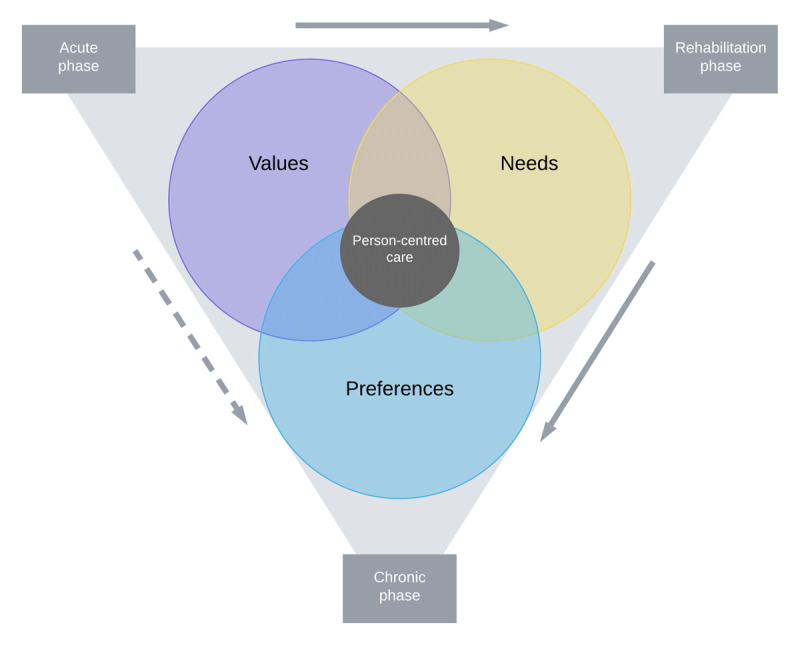
Relationship between patient values, needs and preferences across the continuum of care, based on Van Haitsma et al [22].

In a value-based care system, outcomes are measured across the continuum of care and based on what is meaningful to patients [17]. Subsequently, identified care needs are discussed with patients and their caregivers. This personalised (i.e. person-centred) care approach supports active collaboration between patients, families, and providers to create and manage a comprehensive care plan. It requires engaging patients in decision-making and recognising they are unique individuals with their own values, needs and preferences [18]. *Values* can be defined as broad goals that reflect an individual’s consideration of what is important and worthy and that are relevant across different situations [19]. They express what is desirable and influence patients’ needs and preferences. ‘Health care needs’ refer to practices that a patient can benefit from, such as health education, disease prevention, or treatment [20]. From a patient perspective, a *need* can be anything to enhance health and/or comfort. Patient *preferences* are the result of an individual’s evaluation of relevant elements of health care, including anticipated treatments and health outcomes, and how this relates to them [21]. More specifically, a *preference* is an indication of the attractiveness of an option that aims to fulfil a person’s *need*, and is based on one’s *values* (see Figure 1; based on Van Haitsma et al. [22]).

Understanding patients’ values, their needs and preferences across the continuum of care is essential to make a shift towards person-centred value-based health care. Existing literature on patient values is scarce, particularly with regard to stroke [23,24]. Previous studies about stroke patients’ needs and preferences have focused on specific aspects of the patient journey, such as the hospital to home care transition, or follow-up care [25,26,27,28]. However, value for the patient is produced by combined efforts of health professionals across the continuum of care. Therefore, there is a need for more knowledge about patients’ needs and preferences in all phases of stroke care including acute care, care transitions, rehabilitation, long term support and reintegration to normal living. Moreover, preferences and needs are determined based on one’s values. Our research contributes to fulfilling the knowledge gap regarding stroke patient’s values. The aim of this study was to explore the values, needs, and preferences of stroke patients across the continuum of care.

## 2. Methods

### 2.1 Study design

We conducted a qualitative semi-structured interview study using inductive thematic analysis [29]. Qualitative research is considered appropriate to acquire an in-depth understanding of patient’s values, needs and preferences [30]. Semi-structured interviews were used to ensure a flexible structure of follow-up questions in exploring patients’ thoughts and experiences [31]. Furthermore, the inductive approach allowed research findings to emerge from the raw data without imposing pre-existing assumptions on the setting under inquiry. This study was conducted in Rotterdam, The Netherlands, as part of the larger ValueCare study [32]. The Information and Communication Technology (ICT) enabled value-based methodology for integrated care (ValueCare) project aims to develop and implement efficient outcome-based, integrated health and social care for older people with multimorbidity, and/or frailty, and/or mild to moderate cognitive impairment in seven sites (Athens, Greece; Coimbra, Portugal; Cork/Kerry, Ireland; Rijeka, Croatia; Rotterdam, The Netherlands; Treviso, Italy; and Valencia, Spain). The Medical Ethics Committee of Erasmus MC University Medical Center in Rotterdam, The Netherlands, declared that the rules laid down in the Medical Research Involving Human Subjects Act (also known by its Dutch abbreviation WMO), do not apply to this research proposal (reference number: MEC-2021-0866).

### 2.2 Setting and selection of participants

Patients were recruited from a single-site large university hospital in Rotterdam, The Netherlands. Electronic patient files were screened to select eligible patients based on the following inclusion criteria: (1) diagnosed with ischemic stroke (first ever or recurrent) within the past 18 months at the time of recruitment; (2) community-dwelling (not in long-term care) at the time of recruitment, and (3) able to provide written consent to participate in this study. Patients who did not speak Dutch were excluded. Purposive sampling was used to include patients with a variety of background characteristics in terms of sex, age, time since stroke and severity of stroke. Eligible patients received an invitation letter by post with information about the study and an informed consent form. Patients who returned the signed informed consent form to the researchers were contacted to plan the interview. All interviews have been conducted by telephone.

### 2.3 Data collection

Patients were interviewed between December 2020 and April 2021 by the first author (EB) and a research student assistant. A topic guide was developed as part of the ValueCare project and adapted to the local context. Interview questions were derived through discussions within the research team (See Appendix for sample interview guide). Before the start of the interview, the interviewer explained that she was a researcher, independent of the clinical team. Informed consent was obtained from all participants. The interview started with asking patients to provide a description of the health care (e.g. treatment, rehabilitation, after care) they received and to reflect on the strong and weak points of received health care. Subsequently, patients were asked to indicate what mattered to them regarding their treatment and how services can be improved. Several follow-up questions were asked to deepen the conversation about patients’ preferences for health care improvements (See Appendix). As part of the interview, patients were asked to fill in a short questionnaire about their characteristics (e.g. living arrangements, perceived health). Interviews lasted between 12 minutes and 38 minutes (24 minutes on average), were audiotaped and transcribed verbatim.

### 2.4 Data analysis

Thematic inductive analysis was conducted [29], using the software program NVivo, version 12. The process of coding was based on the six phases of thematic analysis described by Braun and Clarke [33]: familiarisation with data, generating initial codes, searching for themes, reviewing themes, defining and naming themes, and producing the final analyses. Two researchers (EB, DC) independently read the transcripts. Separately from each other, the researchers applied inductive coding with a focus on experiential claims, particularly with regard to values, needs and preferences. Subsequently, the two researchers discussed initial codes and patterns in the data. Relevant coded data extracts were clustered into potential themes and sub themes. Themes were identified when they appeared consistently in a number of transcripts. Identified themes and sub themes were reviewed and discussed by the research team to ensure they are coherent. If necessary, recoding was performed. The analysis resulted in 5 final themes. A selection of quotes was translated into English using forward and backward translations.

## 3. Results

### 3.1 Sample characteristics

The final sample was formed by 36 participants (15 women and 21 men) of which two-third was aged 70 years or older. Most of the participants had a secondary education level or lower (64%). Among the 36 participants, 26 (72%) had their stroke 12 to 18 months ago. Participant characteristics are further detailed in Table 1.

**Table 1:** Characteristics of patients (*n* = 36).


VARIABLE		*n*(%)

Gender		

	Female	15 (42%)

	Male	21 (58%)

Age		

	50–59	2 (6%)

	60–69	10 (28%)

	70–79	16 (44%)

	80–89	7 (19%)

	90–99	1 (3%)

Education level^a^		

	Primary or lower	1 (3%)

	Secondary or equivalent	22 (61%)

	Tertiary or higher	10 (28%)

	Missing	3 (8%)

Living alone		

	Yes	11 (34%)

	No	25 (66%)

Time since stroke		

	6–12 months	10 (28%)

	12–18 months	24 (72%)

First ever stroke		

	Yes	30 (83%)

	No	6 (17%)

Perceived health		

	Good	16 (44%)

	Fair	15 (42%)

	Poor	2 (6%)

	Missing	3 (8%)


^a^ Highest level of education the participant achieved based on the International Standard Classification of Education (ISCED). ISCED level 0–1 was categorized as ‘primary or lower’; ISCED level 2–5 was categorized as ‘secondary or equivalent’, and; ISCED level 6–8 was categorized as ‘tertiary or higher’ [34].

The themes that emerged from the interviews are described below: (1) patients’ values about health care, (2) information and education, (3) psychological support, (4) follow-up care, and (5) continuity and coordination of care. An overview of the values, needs and preferences of patients based on identified themes and sub themes is provided in Textbox 1.

**Textbox 1:** Values, needs and preferences of stroke patients.


**Values**
Being treated as a unique individual Autonomy Good communicative skills of the professional Compassionate professional Responsive professional

**Needs**
Information regarding the causes, consequences and treatment of stroke Psychological support Proactive follow-up by health care professional Continuity of care

**Preferences**
Tailored information based on individual needs, preferably on paper Counselling by social worker or peer support Timely and coordinated follow-up A familiar face – seeing the same health care professional over time Flexible services and professionals


### 3.2 Theme 1: Patients’ values about health care

#### 3.2.1 Values

This theme includes the aspects that patients valued in health care practices. Five sub themes have been identified: (i) patient uniqueness, (ii) patient autonomy, (iii) communicative skills of the professional, (iv) compassionate professional, and (v) responsive professional.

Some patients expressed the wish to be seen and treated as a unique individual rather than a patient with a health condition.

“It is very pleasant for a patient to be approached and treated as a human being. And not that they [the professionals] are discussing things with someone else about me, while I am in bed and able to talk to them.” P01

Furthermore, patients expected to be involved in their treatment and told that they wanted to be well-informed by the professional. Some patients brought forward that this allows them to feel they are respected and trusted partners in their care.

“If I lie there and cannot move, tell me what you [the professional] are doing. I was not well-informed about what was going on. It is important to engage patients in what you are doing, let them think along.” P36

A simple explanation of their condition and clear information regarding their treatment was also valued by patients. This requires good communicative skills of the professional, described by patients as using plain language, being emphatic and a good listener.

“I prefer to speak to a nurse. A nurse is usually more calm and can explain better in simple language than a physician.” P02

Another key value related to the attitude of the professional was compassion. It was described by patients in many forms, such as a chat, a smile, an act of kindness, or by listening to a patient’s story.

“A listening ear. And not fast, fast, fast. That you have been heard only half and you have to tell your story all over again.” P34

Moreover, patients appreciated to have personal, face-to-face contact with the professional. An understanding professional who is easily approachable and listens sincerely was important to patients.

“It is important that I can tell my story to that person [the professional]. If you feel like we can talk to each other about things, then you can go into depth.” P07

Examples mentioned by patients show that small things can make a big difference in how care is experienced.

“Those small gestures I experienced as very pleasant. Getting a toothbrush is not essential, you can do of course without, but the gesture it symbolised the concern one had. The attention for patients.” P26

Patients expected the professional to be committed and responsive to their expressed needs regarding treatment and care.

“The doctor you visit should listen to your problem and take action. That is important to me, being heard.” P30

### 3.3 Theme 2: Information and education

#### 3.3.1 Need for information about stroke

Patients mentioned the importance of receiving information from their health care professional. More specifically, they expressed the need for information regarding causes of stroke, symptoms and consequences of stroke, treatment decisions, prognosis, and follow-up care.

“You keep asking yourself: how did this happen? My feeling is that I have never had a problem that could cause stroke. I am interested in the causes. I like to discuss this with someone.” P07

#### 3.3.2 Preference for tailored information

Patients wanted information that is specifically tailored to their diagnosis and needs, preferably first hand from their professional and written on paper. Information was used to know what to expect and prepare for during all stages of recovery.

“If a person has had a stroke, the consequences need to be well-explained and what you can expect over time… that you won’t recover the full 100%.” P02

### 3.4 Theme 3: Psychological support

#### 3.4.1 Need for psychological support

After discharge, psychological support was indicated by some patients as needed but often lacking for patients and their families. Attention for the psychosocial impact of stroke during follow-up consultations with health care professionals was important to patients. Patients reported to be more emotional than they used to be. Feelings of anxiety and a lack of confidence were mentioned.

“In the beginning I had terrible fear, because you just walk on the street and can collapse. You no longer trust your body. I live alone, and yes, I fear that something like this would happen again.” P15

#### 3.4.2 Preference for counselling or peer support

Some patients also experienced feelings of anger and frustration. Having someone with whom to discuss difficulties was suggested by patients to cope with emotional changes after stroke. Examples mentioned were counselling by a social worker or peer support.

“Physically, I think I am doing okay. [….] But my head is full of weird things. I am easily angry, sad, emotional. And I would have liked to talked to someone about that.” P20

### 3.5 Theme 4: Follow-up care

#### 3.5.1 Need for proactive follow-up by health care professional

We defined follow-up care as care after a stay in the hospital or rehabilitation clinic. A few patients received follow-up care from a community stroke nurse; they appreciated it and considered it helpful. Some patients were dissatisfied with a lack of proactive follow-up from their GP, the hospital, or allied health care professionals. Most of them expressed they received limited follow-up care or not well dosed.

“I actually had to arrange everything myself, for example, home care and physiotherapy. I mean all of that did happen in the end, but the follow-up care from the hospital, I just think that is very bad.” P25

In some cases, patients experienced the relationship with their GP to be difficult. They expect their GP to be informed about their situation and felt disappointed by the lack of follow-up.

#### 3.5.2 Preference for a timely and coordinated follow-up

In general, patients expressed the wish to have more frequent and coordinated follow-up consultations by their health care professional to check how they are doing.

“Of course she [GP] received all information from the hospital. Even if it was a bit delayed, she could still have called.” P29

### 3.6 Theme 5: Continuity and coordination of care

#### 3.6.1 Need for continuity of care

All patients indicated the need for continuity of care – an ongoing relationship with the same health care professional. Patients experienced failures in coordination particularly at points of transition which required shifts of responsibilities and information flow.

#### 3.6.2 Preference for a familiar face

Patients preferred a professional that knows about their condition and does not need reminding.

“In my view, the communication between doctors is very poor. I have to continuously tell what is wrong with me, while they can look in my health record, including the pharmacist. They all have permission.” P02

#### 3.6.3 Preference for flexible services

Preference is given to services and professionals that are flexible and able to adapt to the needs of patients over time.

“When you get home you need the most help. And over time, after a few weeks, that will be less. And they [home care organisation] turn it right around. They give minimal help, and they say if you need help, let us know but that can take up to eight weeks.” P32

## 4. Discussion

This study aimed to explore the values, needs, and preferences of stroke patients across the continuum of care. The interviews yielded a deeper understanding of the long-term impacts of stroke on patients and provide input to enhance person-centred stroke care. Five themes emerged from the analysis: (1) patients’ values about health care, (2) information and education, (3) psychological support, (4) follow-up care, and (5) continuity and coordination of care.

The aspects that patients valued in health care practices were mainly related to the skills and attitudes of professionals, such as good communicative skills and being compassionate. Compassion can be described as the ability of the professional to understand a person’s suffering and the willingness to promote the wellbeing of that person [35]. Consistent with previous studies, patients valued a concerned professional who is easily approachable and communicates clearly [23,24,35]. This is particularly important regarding providing information to patients. In a qualitative study by Martin-Sanz et al. [36] among stroke patients, listening, asking questions to patients, and not being in a hurry was associated with being a good health care professional. Yet, traditional fee-for-service payment models, in which different providers are paid separately for their services, provide little or no reward for delivering optimal stroke care and enhanced coordination of care [16].

Patients expressed the need for clear written information tailored to their diagnosis and needs. Tailoring information can enhance person-centred communication which is associated with increased patient participation [37]. Furthermore, patients preferred a simple explanation of their condition. This requires adequate communication skills of professionals to be able to recognise patients with low health literacy [38]. Previous studies have reported that information can be difficult to assess for stroke patients if language is too medical [6,28]. It is recommended to use short sentences, to define technical terms and to use visuals which represent older people in a positive way [39].

One of the strengths of this study is that patients were asked to elaborate on their experiences with care from hospital to home. Stroke patients emphasised the need for psychological support after discharge. Frustration, anger management issues, emotional lability, and anxiety were commonly experienced. This stresses the need for early and easy accessible psychological services including peer support, as well as support for informal caregivers because of the impact of emotional distress on the close environment [5]. Improved coordination between health care providers across the care continuum, such as neurologists, GPs, social workers, and psychologists, can facilitate the identification of unmet psychological needs [6].

With respect to follow-up care, patients had mixed experiences. Follow-up visits by a community stroke nurse were experienced positively by patients. However, patients also reported to be dissatisfied with a lack of proactive follow-up from their GP or hospital. They expected to have more frequent and coordinated follow-up visits once they were home. Similar to our findings, studies on the experiences of stroke survivors show patients often feel abandoned post-discharge [6,28,40]. In a qualitative study by Lindblom et al. [27] stroke patients describe a lack of active involvement and dialogue around their transition from hospital to home. Consequently, patients may feel that the responsibility for their own care is forced upon them without support or preparation. This links to a need among patients for continuity of care which was defined as a continuous relationship with a health care provider.

Continuity of care includes pre-discharge and post-discharge activities, such as discharge planning, provision of adequate information and education, and timely communication between health care providers [11]. According to patients, this process requires improvement, as they experienced a lack of communication among health care providers, which adversely affects the continuity and coordination of care. Consistent with the literature, a trusted relationship between the patient and health care professional is crucial in order for patients to feel secure, especially during the period shortly after discharge [36,41]. The findings of this study emphasised the need for long-term support in the chronic phase of care to address the social and functional determinants of recovery.

A limitation of this study was that patients were approached by letter to ask them if they were willing to participate in the study. This may have led to selection bias, oversampling those patients with critical comments and an underrepresentation of vulnerable patients. However, our sample also included patients with poor perceived health, therefore this appears not to be a major problem. In addition, the results of this study might be impacted by the COVID-19 pandemic as this affected face-to-face patient-provider communication which was emphasised by patients as an important aspect in their care experience. Furthermore, the study was performed within the specific context of the Netherlands, therefore insights might not be transferable to other settings. To increase the generalisability of our findings, we have reached variation in our sample in terms of patient characteristics (e.g. sex, age, severity of stroke). Moreover, this study includes a high number of patient perspectives resulting in a rich data set and adjudication of the analysis was done through consensus coding. A strength of this study is that our qualitative approach, using a sound methodology, allowed broad insight into values, needs, and preferences of patients regarding the full trajectory of stroke care instead of focusing on certain aspects of care. This manuscript integrates concepts from multiple disciplines which cannot be translated one-on-one to interviews. Instead patients were asked what mattered to them regarding treatment and care (values), how services can be improved (needs), and more specifically how needs could be met (preferences). Our sample included relatively few participants with low literacy. To engage patients with low literacy better, visual and artistic methods can be used, for example, asking participants to respond to pre-selected images related to the topic of interest [42]. Future research is recommended to explore how the values, needs and preferences of patients can inform practice guidelines and ultimately improve care delivery.

Current (post-)stroke care practices are variable and do frequently not address patients’ individual needs. Results of this study imply that value-based care for stroke patients can be improved by taking a more personalised approach. Recognising patients’ values is a key element to move towards personalised (i.e. person-centred) care [18]. Further research is needed to validate and enrich our findings on patient values. Our results also indicate a need for tailored information provision and improved communication skills of professionals. Shared decision-making tools can promote a patient’s knowledge and satisfaction by enhancing patient participation [43]. In addition, mobile health applications can be used to facilitate tailored information provision for patients [44]. Moreover, health technologies that facilitate data sharing and support health care delivery have the potential to improve communication between health care providers [45]. In combination with a community stroke nurse, who supports patients individually and coordinates access to required stroke services, this can enhance the continuity of care [46]. Future research is recommended to explore the role of the community stroke nurse in improving the continuity and coordination of stroke care.

## 5. Conclusions

The aspects that stroke patients valued in health care practices were mainly related to the skills and attitudes of professionals. Stroke patients emphasised the need for tailored information and education, psychological support, follow-up visits, and improved continuity and coordination of care. It is advocated for professionals in stroke care to adopt a more personalised care approach, in order to satisfy the individual needs of patients with regard to information, communication and follow-up care. The findings of this study provide insight into the values, needs and preferences of stroke patients to adopt person-centred value-based health care.

## Appendix: Sample Interview Topic Guide

Introduction

- Introduction with background of the study, aims and structure of the interview.
- Check for provision of informed consent and permission for audio-taping.

Experiences and needs

- What is/was going well, considering all health and social care you currently receive/have received in relation to your stroke treatment?
- What is/was not going so well, considering all health and social care you currently receive/have received in relation to your stroke treatment?
- How can stroke services across the continuum of care (hospital, rehabilitation and at home in the community) be improved?

Values and preferences

- According to you, what is important and/or valuable for stroke patients regarding treatment and care?

Closing

- Additional topics raised by the patient.
- Thank you statement and closing.
